# Development of a comprehensive measure of organizational readiness (motivation × capacity) for implementation: a study protocol

**DOI:** 10.1186/s43058-020-00088-4

**Published:** 2020-11-11

**Authors:** Timothy J. Walker, Heather M. Brandt, Abraham Wandersman, Jonathan Scaccia, Andrea Lamont, Lauren Workman, Emanuelle Dias, Pamela M. Diamond, Derek W. Craig, Maria E. Fernandez

**Affiliations:** 1 Department of Health Promotion & Behavioral Sciences, Center for Health Promotion and Prevention Research, University of Texas Health Science Center at Houston School of Public Health, 7000 Fannin St., Houston, TX 77030 USA; 2 Health Promotion, Education, and Behavior, Arnold School of Public Health, University of South Carolina, 915 Greene Street, Columbia, SC 29208 USA; 3 Wandersman Center, 1512 Laurel St., Columbia, SC 29201 USA; 4 The Dawn Chorus Group, 1014 Hartman Road, Reading, PA 19606 USA; 5 Core for Applied Research and Evaluation, Arnold School of Public Health, University of South Carolina, 220 Stoneridge Drive, Suite 103, Columbia, SC 29210 USA

**Keywords:** Readiness, *R* = *MC*^2^, Interactive systems framework for dissemination and implementation, Implementation measurement, Colorectal cancer screening implementation

## Abstract

**Background:**

Organizational readiness is important for the implementation of evidence-based interventions. Currently, there is a critical need for a comprehensive, valid, reliable, and pragmatic measure of organizational readiness that can be used throughout the implementation process. This study aims to develop a readiness measure that can be used to support implementation in two critical public health settings: federally qualified health centers (FQHCs) and schools. The measure is informed by the Interactive Systems Framework for Dissemination and Implementation and *R* = *MC*^2^ heuristic (*r*eadiness = *m*otivation × innovation-specific *c*apacity × general *c*apacity). The study aims are to adapt and further develop the readiness measure in FQHCs implementing evidence-based interventions for colorectal cancer screening, to test the validity and reliability of the developed readiness measure in FQHCs, and to adapt and assess the usability and validity of the readiness measure in schools implementing a nutrition-based program.

**Methods:**

For aim 1, we will conduct a series of qualitative interviews to adapt the readiness measure for use in FQHCs. We will then distribute the readiness measure to a developmental sample of 100 health center sites (up to 10 staff members per site). We will use a multilevel factor analysis approach to refine the readiness measure. For aim 2, we will distribute the measure to a different sample of 100 health center sites. We will use multilevel confirmatory factor analysis models to examine the structural validity. We will also conduct tests for scale reliability, test-retest reliability, and inter-rater reliability. For aim 3, we will use a qualitative approach to adapt the measure for use in schools and conduct reliability and validity tests similar to what is described in aim 2.

**Discussion:**

This study will rigorously develop a readiness measure that will be applicable across two settings: FQHCs and schools. Information gained from the readiness measure can inform planning and implementation efforts by identifying priority areas. These priority areas can inform the selection and tailoring of support strategies that can be used throughout the implementation process to further improve implementation efforts and, in turn, program effectiveness.

Contributions to the literature

- This study develops a comprehensive measure of organizational readiness in two critical public health settings: federally qualified health centers and schools.
- This study presents an approach to develop pragmatic, yet rigorously tested measures of organizational constructs important to implementation research.
- This study will provide a measurement tool that can be used throughout the implementation process to inform planning and implementation efforts. The tool will also help advance future research by improving the measurement of key implementation constructs.

## Background

Despite the availability of evidence-based interventions (EBIs) for cancer control, EBI implementation and scale-up remain challenging across settings. Implementation science aims to identify and understand factors that influence implementation success so they can be addressed to improve outcomes. Existing gaps in measurement are a major barrier to improving the understanding of such factors and developing strategies to address them. The field has identified organizational readiness as an important factor for implementation success [1–4]. Therefore, a pragmatic organizational readiness assessment can both accelerate and improve dissemination and implementation efforts. This study aims to develop a comprehensive readiness measure in two critical public health settings (federally qualified health centers and schools), targeting two important public health issues (colorectal cancer screening and poor nutrition).

### Organizational readiness

The scientific premise underlying the relation between organizational readiness and implementation outcomes is well established [1–4], and readiness represents a central construct in several implementation science frameworks such as the Interactive Systems Framework for Dissemination and Implementation (ISF) [5], the Consolidated Framework for Implementation Research (CFIR) [6], Getting To Outcomes [7], and Context and Capabilities for Integrating Care [8]. Organizational readiness, as defined across frameworks, is associated with implementation success. The organizational readiness model used in our study consolidates many common constructs shown to be related to implementation success [6, 9–16]. While past studies defined readiness as a construct that refers to an organization’s commitment and collective capability to change [3, 17], and considered it a critical precursor to organizational change, more recent conceptualizations reflect the important role it plays during all phases of implementation [4]. Readiness extends beyond initial adoption and implementation and reflects an organization’s commitment, motivation, and capacity for change over time [18]. This conceptualization of readiness, which emerged from the ISF, is also broader than previous definitions.

**Fig. 1:**
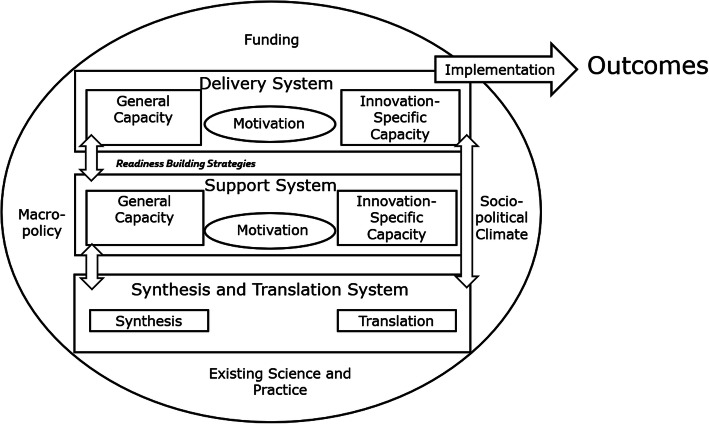
Interactive Systems Framework for Dissemination and Implementation with motivation added. Permission to reproduce the image was obtained from The Journal of Community Psychology. Original source [4]

The ISF is a heuristic for understanding important actions for bridging research and practice across three systems: (1) the *synthesis and translation system*, (2) the *prevention support system*, and (3) the *delivery system* (Fig. 1). The ISF focuses on building the delivery system’s capacity and motivation through the synthesis and translation of research and assistance from the support system. Flaspohler et al. [19] and Wandersman et al. [5] detailed two distinct categories of capacity: innovation-specific and general capacities. Innovation-specific capacities are the knowledge, skills, and conditions needed to put a particular innovation in place. General capacities involve the general functioning of an organization and include the knowledge, skills, and conditions needed to put any innovation into place. Although capacity is a critical component of readiness, it is insufficient for effective change. The model proposes that organizational readiness also depends on the organization’s motivation to implement an innovation [3, 17]. Within the ISF, readiness is a combination of *m*otivation × innovation-specific *c*apacity × general *c*apacity. We abbreviate this as *R = MC*^2^ [4]. Within these three main components, there are multiple subcomponents that make up an organization’s overall readiness (Table 1) [9–11, 19–32].

**Table 1:** Readiness components, subcomponents, and definitions

Component	Subcomponent	Definition
General capacity	Innovativeness	Openness to change in general.
	Resource utilization	Ability to acquire and allocate resources including time, money, effort, and technology.
	Culture	Norms and values of how we do things at our site.
	Climate	The feeling of being part of this site.
	Leadership	Effectiveness of our leaders at multiple levels.
	Staff capacities	Having enough of the right people to get things done.
Innovation-specific capacity	Innovation-specific knowledge and skills	Sufficient abilities to implement the innovation.
	Supportive climate	Necessary supports, processes, and resources to enable the use of the innovation.
	Program champion	A well-connected person who supports and models the use of the innovation.
	Inter-organizational relationships	Relationships between our site and other organizations that support the use of the innovation.
	Intra-organizational relationships	Relationships within our site that support the use of the innovation.
Motivation	Simplicity	The innovation seems simple to use.
	Priority	Importance of the innovation in relation to other things we do.
	Relative advantage	The innovation seems more useful than what we have done in the past.
	Compatibility	The innovation fits with how we do things.
	Trialability	Degree to which the innovation can be tested and tried out.
	Observability	Ability to see that the innovation is producing outcomes.

Reviews of current readiness measures revealed multiple areas for improvement. First, many readiness measures lack rigorous validation approaches [3, 33, 34]. Second, existing measures are designed to be used before implementation rather than for testing readiness over time. Third, no measures assess readiness in a comprehensive manner taking into account critical elements as proposed in the *R* = *MC*^2^ heuristic. A comprehensive readiness measure based on the *R* = *MC*^2^ heuristic is important for all phases of implementation, and it can serve as a useful diagnostic tool for identifying strengths and weaknesses across multiple constructs. Having information about readiness can inform the selection and tailoring of support strategies (e.g., training and technical assistance) throughout implementation to further improve program effectiveness. Overall, readiness is critical for successful implementation and its measurement is essential for research, evaluation, and pragmatic assessment.

Previously, our team developed a readiness measure based on the ISF and the *R* = *MC*^2^ heuristic to assess readiness for implementing a health improvement process among community coalitions. Existing quantitative and qualitative data provided preliminary support for the reliability and validity of the readiness measure in various settings. Additionally, the readiness measure has gained substantial interest from a range of organizations both in the USA and internationally, and in both the research and practice communities. Despite high levels of interest, more work is needed to rigorously develop, adapt, and test the readiness measure across settings. Thus, an important aspect of our work is to further develop the readiness measure for use in federally qualified health centers (FQHCs) to target colorectal cancer screening (CRCS), and for use in schools to target nutrition programs.

### Implementation of EBIs to increase colorectal cancer screening in clinical settings

The US Preventive Services Task Force recommends CRCS to begin at age 50 for average-risk individuals using recommended tests [35]. From 2000 to 2015, CRCS rates increased, but overall, CRCS remains vastly underutilized in comparison to other types of cancer screening [36–38].

CRCS rates fall below the *Healthy People 2020* national goals of 70.5% (Objective C-16) and National Colorectal Cancer Roundtable goal of 80% in every community [16]. Current CRCS rates in the USA are 63.3% with significant variability across race and ethnicity, age, income, education, and regular access to care [37, 38]. Recently, CRCS efforts have been interrupted and rates have significantly decreased due to additional challenges resulting from the COVID-19 pandemic [39, 40].

In 2010, CRCS was added to the clinical quality measures required by health centers, making improving CRCS rates a prominent focus of FQHC’s efforts. To help increase CRCS rates, FQHCs look to EBIs, such as those suggested by the Centers for Disease Control and Prevention (CDC) Colorectal Cancer Control Program (CRCCP), which has prioritized four EBIs: provider assessment and feedback, provider reminders, client reminders, and reducing structural barriers. The CDC CRCCP views these as the most feasible interventions for implementation in FQHC and other primary care settings [41, 42]. Despite the focus on CRCS in FQHCs and through programs such as the CRCCP, the implementation of these approaches has been slow and inconsistent [41–45]. Therefore, there is a need to better understand FQHC’s readiness for implementation through a validated comprehensive measure.

### Implementation of EBIs to improve nutrition in schools

Implementation of school-based programs to improve poor nutrition remains a public health priority. In 2013, there were an estimated 7.8 million premature deaths worldwide that could be attributable to inadequate fruit and vegetable (F&V) intake [46]. Low intake of F&V increases risk of childhood obesity, type 2 diabetes, cancer, cardiovascular disease, and all-cause mortality [47–49].

Furthermore, 60% of US children consume fewer fruits and 93% consume fewer vegetables than recommended [50]. While EBIs for F&V consumption in children exist [51–54], additional work is needed to improve the implementation of these approaches in schools. In this study, the research team aims to test the readiness measure with a school-based program with ongoing implementation. This program, titled Brighter Bites, is an EBI to improve F&V intake among low-income children and their families [51, 55, 56]. The program has shown rapid dissemination from one school in 2012 to more than 100 schools in Texas, with more than 20,000 families participating. While existing dissemination efforts are encouraging, implementation varies widely across sites. Organizational readiness is likely one of the factors that influence the implementation of this program. Thus, having a comprehensive, valid, and reliable readiness measure for schools could (1) help understand implementation variability and (2) help lead to greatly enhanced implementation efforts and broad program scale-up. Overall, the use of a consistent readiness measure across settings and topic areas can advance implementation science and further our understanding of how organizational readiness influences implementation outcomes.

### Aims

The overall goal of this study is to develop a theoretically informed, pragmatic, reliable, and valid measure of organizational readiness that can be used across settings and topic areas, by researchers and practitioners alike, to increase and enhance the implementation of cancer control interventions. The aims of this study are to (1) adapt and further develop the current readiness measure to assess readiness for implementing EBIs for increasing CRCS in FQHCs; 2) test structural, discriminant, and criterion validity of the revised readiness measure as well as reliability by assessing scale reliability, temporal stability, and inter-rater reliability; and 3) adapt and assess the usability and validity of the readiness measure in the school setting for implementing a nutrition-based program.

## Methods

**Fig. 2:**
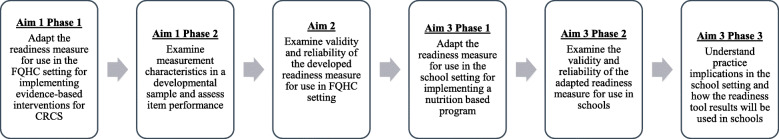
Study flow diagram. FQHC, federally qualified health center; CRCS, colorectal cancer screening

There are multiple phases for this study. Figure 2 outlines the aims and respective phases. Aims 1 and 2 will develop and test the validity of the readiness measure in FQHCs. Aim 3 will adapt and test the validity of the measure in schools.

### Aim 1 phase 1: adapt the readiness measure for use in the FQHC setting

For aim 1 phase 1, we will conduct a qualitative analysis in nine health center sites from varying FQHC systems in Texas and South Carolina. The research team will carry out semi-structured group and individual interviews with health center employees from different job types (medical/clinical assistants, nurses, providers, and quality improvement staff/clinic managers). We will conduct about five group interviews with up to five participants in each interview. We will also conduct about 15 individual interviews. Both the group and individual interviews will ask about factors impacting previous practice changes, current CRCS efforts, how readiness relates to implementation, and methods to collect readiness data from health centers.

After completing group and individual interviews, the research team will conduct a series of cognitive interviews, which is a method commonly used to improve survey questions [57]. These interviews will ask participants about their understanding of specific items included in the readiness measure and what they need to think about when responding to respective items. We plan to initially complete 10 interviews (five in South Carolina and five in Texas) and conduct additional interviews as needed. All interviews will be recorded and transcribed verbatim for analysis.

#### Recruitment and analysis

We will work with existing contacts from FQHC sites in South Carolina and Texas to recruit interview participants. Individual and group interviews will last 30–60 min and cognitive interviews will last 60–90 min. All participating FQHCs will receive a summary describing our findings from the adaptation phase and receive updates about future developments with the readiness measure. The research team will conduct a rapid qualitative assessment to examine readiness constructs in the clinic setting using a matrix analysis approach [58, 59]. The team will also conduct a content analysis using iterative deductive codes to identify readiness constructs discussed during the interviews [60–64]. For the content analysis, two team members will open code transcripts (inductively) to identify prominent, emergent themes. Collectively, this information will be used to make edits to the readiness measure and inform how the measure will be distributed in health center sites.

### Aim 1 phase 2: examine measurement characteristics in developmental sample

The objective of aim 1 phase 2 is to conduct development testing of the readiness measure. This process will begin by administering the expanded version of the readiness measure (includes all questions in the item pool) to a development sample of staff at FQHCs. The overall goal of this step is to reduce the number of items for each readiness construct to produce pragmatic scales for readiness subcomponents that most efficiently assess each construct.

#### Recruitment strategy and data collection

We will recruit a sample of up to 100 health center sites by working with existing FQHC system networks, Primary Care Associations, and umbrella organizations for community health centers in Texas and South Carolina. We will also engage with existing partners including the American Cancer Society, National Colorectal Cancer Round Table, and Southeastern Consortium for Colorectal Cancer to help recruit clinic networks. Within each participating health center, we will work with a health center contact to help recruit FQHC staff to complete the readiness survey. Staff eligibility will be based on quotas to capture an approximately equal number of respondents from each job type (4 medical/clinical assistants, 3 nurses, and 3 physicians/nurse practitioners/physician assistants). Thus, up to 10 surveys will be completed by each health center site with a relatively equal distribution of respondents between the targeted job types.

The designated contact at each health center site will help distribute the survey electronically to their respective health center site staff using a REDCAP survey link. The survey will include questions about individual demographic information and the readiness subcomponents. The research team will send reminder emails to the FQHC contact 2 and 4 weeks from the initial distribution to support data collection efforts. Both the health center site and individual respondents will receive incentives for participating.

#### Survey development analyses

The survey development analysis will consist of three steps: (1) assess item characteristics, (2) assess relations between items, and (3) examine latent factor structures for each subcomponent. For step 1, we will examine descriptive statistics for each question (means and distributions) to provide information about the range of responses and whether there is evidence of floor or ceiling effects (majority answers falling on one end of the scale). We will also examine how much variance is explained by the health center site level for each item using intraclass correlation coefficients (ICC(2)). For step 2, we will assess correlation matrices and corrected item-total scale correlations for each readiness subcomponent at the individual- and site-levels.

For step 3, we will examine the underlying factor structure for each readiness component using multilevel confirmatory factor analysis (CFA) models. As a preliminary step, we will conduct CFA models for each readiness component (general capacity, innovation-specific capacity, and motivation) using individual level data. We will examine modification indices to highlight potential points of model strain. We will then conduct a series of multilevel CFA models for each readiness subcomponent. In multilevel models, we will note items with low factor loadings (< 0.40). After assessing each subcomponent separately, we will conduct a series of multi-factor multilevel CFA models for highly related subcomponents. We will determine highly related subcomponents by using the correlations between subcomponents from the first set of CFA models and by assessing the site-level correlations between subcomponents. If correlations between two subcomponents are > 0.80, we will conduct a multi-factor multilevel CFA model with both subcomponents included to identify potential overlap between subcomponents and items.

For factor models, we will assess models using multiple indices of fit: chi-square (non-significant implies good fit of the model to the data), comparative fit index (CFI, > 0.95 = good fit), Tucker-Lewis index (TLI, > 0.95 = good fit), standardized root mean square residual for both the site and individual levels of the model (SRMR, < 0.05 = good fit), and root mean square error of approximation (RMSEA, < 0.05 = good fit). To address missing data and the potential for non-normal distributions for items, we will use full information maximum likelihood (FIML) estimation with robust standard errors. Using the collective information from the descriptive statistics, item correlations, and CFA models, we will identify the most relevant and high performing items for each readiness subcomponent. Given the many subcomponents included in the readiness measure, we aim to include 4–7 items per subcomponent. This number is thought to be ideal to establish a balance between brevity and reliability/validity [65]. Once the items are established for each subcomponent, we will assess the scale reliability for each subcomponent using a multi-level CFA-based approach [66].

#### Sample size for aim 1 phase 2

The sample size for phase 2 is driven by multilevel, CFA models, which generally require large samples to produce stable results. Generally, having a larger sample size will decrease sampling error variance, which can help lead to more stable solutions [67]; however, sample size adequacy cannot be entirely known until analyses themselves have been conducted [68]. Therefore, our proposed sample size is based on existing recommendations, recruitment feasibility, the complexity of the proposed models, and past experience. Guidelines suggest when using a multilevel factor analysis approach, it is important to have 50–100 groups (or health center sites) [69].

Based on these recommendations, we plan to recruit 100 sites with up to 10 respondents per site leading to a total of 1000 individual respondents. To determine the lower bound estimate for the sample required, we calculated the number of parameters estimated for various types of models. Having a sample of 100 sites, with 1000 individuals will be adequate for testing models with up to 19-items, which is statistically sufficient and an attainable recruitment goal.

### Aim 2: examine validity and reliability of the developed readiness measure

Aim 2 focuses on examining the validity and reliability of the readiness measure using a different sample of health center sites to ensure the measure is generalizable beyond the sample used to develop it. For this aim, we will recruit a new sample of sites and respondents to test the final factor structures established in aim 1 phase 2. We will also conduct other forms of validity and reliability testing.

#### Recruitment strategy and data collection

We will use the same recruitment approach explained in aim 1 phase 2 by working with existing networks and partnerships to recruit a new sample of health center sites. We will also use the same data collection procedures explained in aim 1 phase 2. In addition to the newly developed readiness measure, we will include additional questions on the survey to assess implementation outcomes, which will be used for criterion validity.

#### Validity and reliability analyses

##### Multilevel CFA models

We will conduct a series of multilevel CFA models to validate the readiness measure developed in aim 1 phase 2. First, we will test multilevel CFA models for each readiness subcomponent separately to reduce the number of model parameters. Factor loadings will be allowed to freely estimate in these unrestricted models. We will then empirically test whether the factor structures are the same between the individual and site levels of the model. Thus, we will conduct a second set of models where factor loadings are constrained to be equal across the site and individual levels. We will compare model fit for corresponding constrained and unconstrained models using Satorra-Bentler’s scaled chi-square difference tests [70]. Results indicating equal or better fit for constrained models will provide evidence for similar factor structures between the individual and site levels and further support aggregation of individual scores to represent sites. Similar to aim 1 phase 2, we will use FIML estimator to account for missing data and the standard fit indices to assess model fit. Models with good fit that contain items with strong factor loadings (> 0.60) [71] will provide evidence for structural validity.

#### Discriminant validity

We will evaluate discriminant validity by examining correlation coefficients between subcomponents using individual level data and aggregated data by sites. Because these correlations will be used as a preliminary screening to identify potentially overlapping subcomponents, we will use a more conservative correlation coefficient value of > 0.70 to suggest there may be overlap between readiness subcomponents. We will further assess highly correlated pairs of readiness subcomponents by conducting multilevel CFA models. Fitting these models will help determine the level of correlation when factoring out the error and further identify where potential measurement overlap occurs (at the individual level, site level, or both levels). Correlations between subcomponents within multilevel CFA models > 0.80 will suggest overlapping constructs and poor discriminant validity [71, 72].

#### Criterion-related validity

To evaluate criterion-related validity, we will assess readiness subcomponents concurrently with implementation outcomes and then assess their relationship, both within and across sites. Implementation of CRCS EBIs will be assessed using a self-report measure completed by site staff [41, 45]. Assessing the relation between readiness and implementation outcomes calls for multilevel modeling, where given one outcome that is to be predicted, we model (a) differences in the mean outcome across sites, (b) site-level predictors of the mean outcome, (c) differences across sites in their regression lines (i.e., variance in the intercepts and slopes for predictors of the outcome), and (d) site-level predictors of both intercept and slope differences. In these multilevel regression models, EBI implementation levels will be the outcomes, readiness subcomponent scores will be independent variables, and site characteristics will serve as control variables.

#### Scale reliability

Reliability refers to the consistency of measurement and has been defined as the ratio of a scale’s true-score variance to its total variance [66, 72]. We will assess scale reliability for each readiness subcomponent using a multilevel CFA-based approach [66]. Using this approach, each subcomponent’s true-score and error variance will be calculated based on CFA estimates of factor loadings and residual variances at each respective level [66].

#### Temporal stability

We will assess temporal stability to determine the consistency of readiness scores over a stable period of time. We will use a test-retest approach where a subsample of 30 health center sites will complete the readiness assessment 1–2 weeks apart. We will test the reproducibility of results by computing ICCs using a two-way mixed effects model [73, 74], which is preferred when testing scores rated by the same respondents [75]. We will assess ICCs using both individual and site level data where values > 0.70 will be used to indicate consistent measures.

#### Interrater reliability

We will test interrater reliability to determine the relative consistency of participant responses within respective sites. Interrater reliability estimates will help determine whether participants within sites provide consistent relative rankings for readiness questions [75]. For assessing reliability, we will compute ICC(1) and ICC(2) using one-way random effects ANOVA [76]. ICC(1) will provide an estimate of variance explained by health center site where larger values indicate shared perception among raters within sites. ICC(2) indicates the reliability of the health center site level mean scores and varies as a function of ICC(1) and group size. Larger ICC(1) values and group sizes will lead to greater ICC(2) values indicating a more reliable group mean score [75].

#### Sample size of aim 2

The sample size for aim 2 is driven by the multilevel CFA models. We consider the same factors from aim 1 phase 2 to be relevant in determining the sample size for aim 2. We expect the multilevel CFA models will contain fewer items in aim 2 (vs. aim 1 phase 2) because some items will be eliminated during development. Therefore, we will have an adequate sample to support our proposed multilevel CFA approach. Our test-retest sample is based on recruitment feasibility and the fact that test-retest reliability is traditionally and effectively conducted in smaller samples [77], where a sample size of 30 is sufficient.

### Aim 3 phase 1: adapt the readiness measure for use in schools

The purpose of aim 3 phase 1 is to adapt the readiness measure to be appropriate for use in schools implementing health promotion programs. We will use a qualitative approach to identify elements of the measure that need to be adapted (e.g., edits to question phrasing). Specifically, we will work with an existing network of schools participating in the Brighter Bites program, an EBI to improve fruit and vegetable intake among low-income children and their families. We will conduct a series of group and individual semi-structured interviews with different members of school staff involved with Brighter Bites implementation. Interviews will include questions about the readiness measure and its use for schools. We will recruit study participants with the help of Brighter Bites school contacts. Interviews will last 30–60 min, and participants will receive an incentive. Similar to aim 1 phase 1, we will carry out a content analysis using iterative deductive and inductive codes.

### Aim 3 phase 2: examine the validity and reliability of the adapted readiness measure

Aim 3 phase 2 will test the validity of the readiness measure in the school setting. For this phase, we will follow a similar approach to aim 2. We will work with the Brighter Bites network of schools to obtain a validation sample from over 100 participating schools in Texas. The Brighter Bites program distributes an annual electronic survey to schools and thus the adapted readiness items will be included in this survey. The survey will be completed by school staff who are involved with program implementation. The number of respondents per school will be limited to five with a maximum of three teachers or three administrators (whichever job type is first to reach three). This approach is to reduce the burden on schools and to maximize the number of schools rather than the individual respondents, which will allow for more stable multilevel models. We will follow a similar validation procedure outlined in aim 2 with the exception of not assessing test-retest reliability.

#### Sample size of aim 3 phase 2

A sample of 80 schools with 5 respondents per school (leading to 400 individuals) will be an adequate sample for validity and reliability tests. As previously discussed, we expect 4–7 items per readiness subcomponent in the final readiness tool. Therefore, a sample size of 80 schools will be adequate to test 1-factor and 2-factor models with up to 15 items.

### Aim 3 phase 3: understand the use of readiness results in the school setting

To gain a better understanding of how measure results are used in schools, we will conduct a second set of group and individual interviews with the same schools and methods as previously described. These interviews will focus on how schools use scores from the readiness measure to make mid-course corrections to implementation processes. The interviews will also inform recommendations for using the readiness measure to support ongoing implementation efforts.

## Discussion

There are few detailed descriptions of implementation science studies that focus on the development of organizational measures using multilevel approaches. The approach of this study will support the development of pragmatic, yet rigorously validated measures for readiness that can be used to enhance implementation in multiple settings. This is essential because while understanding the relative importance of readiness for implementation can move the field forward, the use of pragmatic measures to improve implementation practice can help fill the research to practice gap. The developed readiness measure is designed to be relevant for both pre-implementation and during implementation and will serve as a useful diagnostic tool for identifying strengths and weaknesses across multiple components and subcomponents of readiness. Further, the adaptation approach used in this study will provide a blueprint for future researchers and practitioners who want to assess readiness in populations and settings beyond FQHCs and schools.

The information gained from a comprehensive readiness assessment can help implementation efforts in many ways. For example, the readiness assessment can serve as an initial step within a readiness building system to improve implementation outcomes. By completing the readiness assessment, the results can inform planning and implementation by (1) guiding internal discussions within an organization and (2) helping identify which subcomponents to prioritize with readiness building strategies. From these prioritizations, an organization can form a targeted plan to improve readiness and implementation of an EBI. The plan can help provide the underpinning for who needs to do what within an organization and how selected theory-based change methods can improve implementation. Further, the readiness assessment and plan can be revisited and refined throughout the implementation process to continue to build and maintain implementation readiness in settings that are inherently dynamic.

There are multiple practical and operational challenges involved in performing this study. First, we are working with many different FQHCs across the USA to collect both qualitative and quantitative data. This requires a recruitment effort that builds on existing relationships and expands networks for collaborative work. For this, we plan to use principles from participatory research by creating partnerships that have value to participating organizations. Specifically, we will share back results from the readiness assessment in an actionable format with partners. This format will include information about readiness-building priority areas (based on assessment results) along with suggested next steps and guidance for how to target these priority areas.

Another study challenge is conducting research during the COVID-19 pandemic. During this time, FQHCs have had to implement urgent practice changes to provide COVID-19 related services and maintain ongoing services in a safe manner. This not only impacts FQHCs’ willingness and ability to partner for research, but it also influences how the research can be conducted. To address these challenges, we have been communicating with FQHC partners to offer support and help with their ongoing clinic-based efforts, such as aiding in the application for COVID-related grants. In addition, we have adapted research plans to conduct cognitive interviews virtually rather than in-person to protect the safety of participants and research staff. We are also offering flexible solutions to technology and time limitations such as options for virtual or phone interviews, and options to schedule interviews for evenings and weekends (if preferred). We will also work with FQHC partners to administer surveys electronically (as originally planned). When the study transitions to collaborating with school partners, we will monitor challenges in the school setting and use a flexible research approach similar to our approach with FQHCs.

### Summary

This study outlines a comprehensive approach to develop and validate a measure of organizational readiness. The approach is informed by a rigorous scale development process [78] and addresses the multilevel nature of the readiness assessment. In addition, the approach is designed to create a measure that has strong psychometric properties and yet can also be adapted to be appropriate across settings and topic areas. Collaborating with FQHC and school partners during this unique time presents added challenges for this work. Our team will document these challenges along with our solutions throughout the study, which can help inform future collaborative efforts.

## Data Availability

Not applicable
